# Does *HER2*/*neu* overexpression in breast cancer influence adjuvant chemotherapy and hormonal therapy choices by Ontario physicians? A physician survey

**DOI:** 10.3390/curroncol13010001

**Published:** 2006-02

**Authors:** J.A. Myers, G. DeBoer†, E. Warner

## 1. INTRODUCTION

The *HER2* gene (formerly called c-*erbB*-2) encodes a 185-kDa transmembrane glycoprotein with intra-cellular tyrosine kinase activity. In breast cancer, overexpression of *HER2* is seen in 20%–30% of breast cancer cases 1. Initially, the goal of *HER2* status assessment for breast cancer was to determine which patients with metastatic disease might benefit from treatment with trastuzumab (Herceptin: Genentech, San Francisco, CA, U.S.A.), the mono-clonal antibody to *HER2*, either alone or in combination with chemotherapy. Subsequently, the *HER2* status of breast tumours was shown to have a potential role in the selection of adjuvant systemic therapy because of prognostic relevance and a putative role in predicting resistance to specific chemotherapies and hormonal therapies. Accordingly, some centres routinely tested for tumour *HER2* status at the time of diagnosis.

At the 2005 meeting of the American Society of Clinical Oncology, early reports from three randomized studies demonstrated a 50% improvement in disease-free survival and a 33% improvement in overall survival with the addition of trastuzumab to standard adjuvant chemotherapy. Thus, it is now essential that *HER2* status be available at the time of breast cancer diagnosis. Although medical oncologists will use this information to determine the need for adjuvant trastuzumab, the extent to which *HER2* status might affect their adjuvant chemotherapy and hormonal therapy recommendations is not known.

Amplification of *HER2* has been found to correlate with a worse prognosis in both node-negative and node-positive disease 2–4. However, whether patients with such amplification would benefit from receiving more aggressive systemic therapy than they might otherwise receive is still unknown. Reports about the ability of *HER2* overexpression to predict response to systemic chemotherapy and hormonal therapy are conflicting. Studies have suggested that, as compared with patients without *HER2* overexpression, patients with such overexpression benefit less from chemotherapy regimens that lack an anthracycline 5–7. It has also been suggested that patients with *HER2* overexpression are resistant to tamoxifen 8 and that alternative strategies such as aromatase inhibition or ovarian oblation or both may be superior in these patients 9. However, other studies have not found *HER2* overexpression to adversely influence response to tamoxifen 10. Interpretation of the data is further complicated by the fact that large discrepancies exist between centres worldwide with respect to the method of detecting *HER2* gene overexpression 11,12.

Despite the uncertainties, some authors advocated—long before the release of the promising adjuvant trastuzumab data—that, because of its prognostic usefulness, *HER2* testing be routinely performed for all new breast cancer cases 13. In Ontario, the most populous province in Canada, such routine testing was adopted at some centres, but not at others. Accordingly, to better understand whether and how *HER2* status affects systemic chemotherapy and hormonal therapy recommendations by medical oncologists, we conducted a survey of those practitioners across Ontario.

## 2. MATERIALS AND METHODS

An introductory letter, consent form, and questionnaire were mailed to all medical oncologists practicing at cancer centres and teaching hospitals in Ontario, and to all community oncologists across Ontario who were members of the Canadian Association of Medical Oncologists at the time of mailing. Medical oncologists who did not regularly treat breast cancer were excluded. A total of 99 packages were mailed in September 2002.

The questionnaire was divided into two sections:

- Section 1 gathered demographic data, including the physician’s age, years in practice, type of practice, and percentage of time devoted to treating oncology patients in general and breast cancer patients in particular. The questionnaire also asked about the availability to the physician of common prognostic and predictive factors in breast cancer, including tumour size, tumour grade, oestrogen receptor (er) and progesterone receptor (pr) expression, lymphovascular invasion, perineural invasion, lymph node involvement, and *HER2* overexpression.
- Section 2 of the questionnaire was designed in two separate versions (version A and version B). Both versions contained five hypothetical scenarios of newly diagnosed breast cancer cases for which an adjuvant treatment decision was requested. Cases with a risk of recurrence ranging from low to high were included. Each scenario supplied patient demographic data as well as information on the primary tumour, including size, grade, lymphovascular invasion, er/pr status, and lymph node involvement. In each case scenario, *HER2*/*neu* overexpression was also included and listed as positive (*HER2*+) or negative (*HER2*−). Questionnaire versions A and B differed only with respect to the *HER2* status of each case. Version A scenarios 1, 3, and 5 were marked as *HER2*+, and scenarios 2 and 4 as *HER2*−; version B scenarios reversed the *HER2* status of each case. The medical oncologists were randomized to receive either questionnaire version A or version B, with stratification by cancer centre.

The oncologists were blinded to the specific study hypothesis and were told in the letter that the project was evaluating the availability and utility of prognostic and predictive markers for decision-making in the adjuvant treatment of breast cancer. Written informed consent was obtained from all participants.

For each case scenario, physicians were instructed to choose from a list of systemic treatment options one or more regimens that they would recommend. The chemotherapy regimens included were cmf, cef, ac, ac plus Taxol, caf, and mf (see Table I for a description of these regimens—information that was included with the questionnaire). The options of choosing no chemotherapy or an alternative regimen not listed were also provided.

In addition to the systemic chemotherapy options, systemic hormonal therapy options were added for the two hormone receptor–positive cases. The options included were tamoxifen, aromatase inhibitor, ovarian ablation (surgical or medical)—either alone or in combination—and “other therapy.” The questionnaire assumed that some form of hormonal therapy would be offered. Table II shows one sample case scenario from the questionnaire.

For the purpose of analysis, each case scenario was classified as showing a low, intermediate, or high risk of recurrence, based on projected 10-year disease-free survival according to Mayo Clinic criteria 14.

Each physician’s systemic chemotherapy recommendation for a given scenario was grouped into one of three categories: “no chemotherapy,” “less aggressive” (cmf, ac, or mf), and “more aggressive” (ac plus Taxol, cef, fac, or caf). If regimens from more than one category were chosen, the selection was recorded as “less aggressive” because, presumably, the physician would opt to treat the patient with the least toxic of the regimens selected. If no chemotherapy was selected as one of the choices, then regardless of other selections, “no chemotherapy” was recorded. The number of physicians specifically recommending either or both of ac and cmf was noted. For the two case scenarios with hormone-receptor-positive disease, endocrine therapy was coded as either “tamoxifen” or “other.”

All results with ordered categories (that is, “no chemotherapy,” “less aggressive,” and “more aggressive” chemotherapy) were analyzed using chi-square for trend. Variables with two categories were analyzed using the Fisher exact test.

## 3. RESULTS

Of the 99 medical oncologists to whom questionnaires were mailed, 50 received version A (group A) and 49 received version B (group B). The group A physicians returned 30 questionnaires. One questionnaire was incompletely answered; the remaining 29 were included in the final analysis. The group B physicians returned 29 completed questionnaires. Both groups had similar male:female ratios, mean years in practice, and type of practice (Table III). At the time of consultation, *HER2* status was routinely available in 53% and 55% of cases in groups A and B respectively. The other prognostic and predictive factors included in the questionnaire were almost universally routinely available to both groups.

### 3.1 Scenario 1

**Figure 1:**
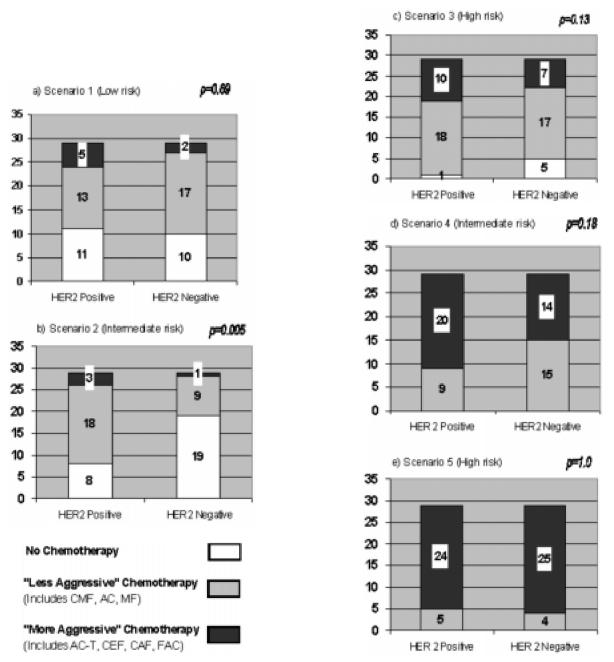
Chemotherapy choices by the surveyed physicians for the case scenarios presented. All tumours were infiltrating ductal carcinoma: a) Age 47, 0.6 cm, grade 3, estrogen receptor negative (ER−), node-negative; b) age 59, 1.5 cm, grade 2, ER+, node-negative; c) age 65, 2.2 cm, grade 2, ER+, node-positive; d) age 43, 1.7 cm, grade 3, ER−, node-negative; e) age 37, grade 2, 1.1 cm, ER+, node-positive.

Scenario 1 presented a 47-year-old woman with a 0.6-cm, grade 3, er/pr-negative, node-negative infiltrating ductal carcinoma (low risk, 90% disease-free survival at 10 years 14). We observed no significant difference in treatment recommendations between the group A and B physicians [Figure 1(a)]. Also, among physicians recommending less aggressive chemotherapy, the proportion of those choosing ac as compared with cmf did not differ by *HER2* status (4/13 vs. 6/17).

### 3.2 Scenario 2

Scenario 2 presented a 59-year-old woman with a 1.5-cm, grade 2, er/pr-positive, node-negative infiltrating ductal carcinoma (intermediate risk, 81% disease-free survival at 10 years). Physicians who received the *HER2*+ version of the scenario were more likely to select some form of adjuvant chemotherapy [21/29 vs. 10/29, *p* = 0.008, Figure 1(b)]. Among the physicians who selected less aggressive chemotherapy, a higher proportion of those receiving the *HER2*+ version recommended ac over cmf (3/9 vs. 3/18), but the difference was not statistically significant (*p* = 0.37).

**Figure 2:**
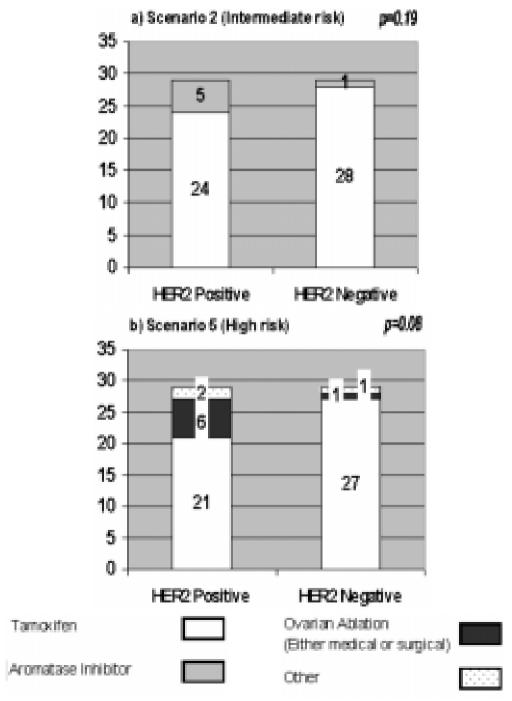
Hormonal therapy choices by the surveyed physicians for estrogen receptor positive (ER+) scenarios. All tumours were infiltrating ductal carcinoma: a) age 59; b) age 37.

For adjuvant endocrine treatment [Figure 2(a)], a tendency to favour aromatase inhibitors over tamoxifen was seen among the oncologists who received the *HER2*+ version (5/29 vs. 1/29, *p* = 0.19).

### 3.3 Scenario 3

Scenario 3 presented a 65-year-old woman with a 2.2-cm, grade 2, er/pr-positive infiltrating ductal carcinoma metastatic to 2 of 11 axillary nodes (high risk, 50% disease-free survival at 10 years). Of the oncologists who received the *HER2*+ version, only 1 of 29 selected “no chemotherapy” as compared with 5 of the 29 who received the *HER2*− version (*p* = 0.13). No statistically significant difference was seen in the aggressiveness of the chemotherapy recommended [Figure 1(c)], nor in the choice of ac over cmf.

### 3.4 Scenario 4

Scenario 4 presented a 43-year-old woman with a 1.7-cm, grade 3, er/pr-negative, node-negative infiltrating ductal carcinoma (intermediate risk, 81% disease-free survival at 10-years). All respondents selected adjuvant chemotherapy regardless of *HER2* status. Of the physicians who received the *HER2*+ version of the scenario, 20 of 29 recommended aggressive chemotherapy as compared with 14 of 29 who received the *HER2*− version [*p* = 0.18, Figure 1(d)].

Among the physicians who selected less aggressive chemotherapy, the proportion of those choosing ac over cmf did not differ by *HER2* status.

### 3.5 Scenario 5

Scenario 5 presented a 37-year-old woman with a 1.1-cm, er/pr-positive, grade 2 infiltrating ductal carcinoma with 2 of 16 nodes positive for cancer (high risk, 56% disease-free survival at 10-years). All respondents selected some form of adjuvant chemotherapy. No difference was seen between group A and B physicians in the recommendation of less aggressive (5/29 with *HER2*+, 4/29 with *HER2*−) versus aggressive chemotherapy [Figure 1(e)], and no significant difference was seen between the two groups in the selection of ac over cmf.

In the group that received the *HER2*+ version of the questionnaire, hormonal treatments other than tamoxifen were more frequently recommended, with 8 of 29 physicians choosing ovarian ablation with or without an aromatase inhibitor in the *HER2*+ group and only 2 of 29 choosing non–tamoxifen based treatment in the *HER2*− group [*p* = 0.08, Figure 2(b)].

### 3.6 Additional Analyses

The data were also analyzed looking exclusively at respondents who indicated that *HER2* testing was routinely performed at diagnosis in their place of practice (32 total, 16 from group A and 16 from group B). We noted statistically significant differences in the chemotherapy recommendations for scenarios 2 and 4, both of which were intermediate-risk cases. In scenario 2, 14 of 16 physicians who received the *HER2*+ version of the question recommended chemotherapy as compared with only 4 of 16 who received the *HER2*− version (*p* = 0.004). In scenario 4, for which all oncologists recommended chemotherapy, 12 of 14 who received the *HER2*+ version of the question selected aggressive chemotherapy as compared with just 6 of 16 who received the *HER2*− version (*p* = 0.009).

## 4. DISCUSSION AND CONCLUSION

The results of our survey suggest that medical oncologists in Ontario use *HER2* status to guide their adjuvant chemotherapy and hormonal therapy treatment recommendations for breast cancer cases with an intermediate risk of recurrence.

For the low-risk case, *HER2* status did not influence chemotherapy selection; however, given that more than 60% of the oncologists recommended chemotherapy in the low-risk scenario, it is conceivable that *HER2* status might have a greater impact even on a lower-risk case (that is, similar to the first scenario, but er-positive). Recommendations for the two high-risk cases were not affected by *HER2* status. That finding makes intuitive clinical sense because, for cases already considered high risk based on more traditional prognostic factors, additional information would not be needed to persuade the oncologist to recommend aggressive treatment.

We also saw a trend toward the increased use of hormonal treatments other than tamoxifen for *HER2*+ cases. It is difficult to discern from the survey whether the lack of statistical significance of that trend is an artefact of the modest sample size or a true reflection of varying interpretations of the literature.

The effect of *HER2* status was more pronounced among oncologists for whom *HER2* was routinely available at diagnosis. Presumably, these physicians already routinely incorporated *HER2* status into their decision-making, and those who lacked routine *HER2* information did not. However, it is also conceivable that only the oncologists who felt strongly about the importance of *HER2* status for determining adjuvant treatment would have pushed to have the test routinely performed for all newly diagnosed breast cancer cases at their centre.

One limitation of this study is that our hypothetical cases may or may not accurately reflect the “real world.” However, 170 consecutive charts of newly diagnosed early-stage breast cancer patients at our centre were systematically reviewed by one of the authors (JAM). For cases with a profile resembling any of the study scenarios, the range and frequency of the actual treatment recommendations were similar to those among the survey responses for the corresponding hypothetical scenario.

Although our 60% response rate is above average for most mailed physician surveys 15, it is conceivable that our results may not be easily generalized to the non-responders. However, systematic differences between participants and non-participants with respect to *HER2* status utilization would be unlikely because all potential participants were blinded to the study hypothesis.

More important than the question of whether *HER2* status in breast cancer influences adjuvant chemotherapy and hormonal therapy treatment decision-making is whether *HER2* should influence treatment decision-making at all. The most recent version of Adjuvant! (version 7.0), the popular computer software aid to adjuvant breast cancer therapy decision-making, allows users to insert additional prognostic markers of their own choosing but it does not specifically include *HER2* in the initial profile 16. Sufficiently powered prospective clinical trials in which *HER2* testing methodology is standardized are clearly necessary to clarify whether modifying adjuvant chemotherapy and hormonal therapy according to *HER2* status can favourably alter the natural history of breast cancer. In the meantime, a formal state-of-the-art practice guideline on the use of breast cancer *HER2* status for adjuvant chemotherapy and hormonal therapy decision-making would be extremely helpful to medical oncologists.

## Figures and Tables

**Table I:** Chemotherapy regimens

*Regimen*	*Agent and dose*	*Cycle*
ac	Doxorubicin 60 mg/m^2^ IV, day 1 Cyclophosphamide 600 mg/m^2^ IV, day 1	Every 21 days
ac + Taxol	Doxorubicin 60 mg/m^2^ IV, day 1 Cyclophosphamide 600 mg/m^2^ IV, day 1 then Paclitaxel 125 mg/m^2^, day 1	Every 21 days × 4 Every 21 days × 4
cmf	Cyclophosphamide 100 mg/m^2^ PO, days 1–14 Methotrexate 40 mg/m^2^ IV, days 1 and 8 5-Fluorouracil 600 mg/m^2^ IV, days 1 and 8	Every 28 days
MF	Methotrexate 100 mg/m^2^ IV, days 1 and 8 5-Fluorouracil 600 mg/m^2^ IV, days 1 and 8 Leucovorin 10 mg/m^2^ PO	Every 28 days 1 Hour after methotrexate Starting 24 hours after methotrexate, every 6 h × 6 doses
caf	Cyclophosphamide 100 mg/m^2^ PO, days 1–14 Doxorubicin 30 mg/m^2^ IV, days 1 and 8 5-Fluorouracil 500 mg/m^2^ IV, days 1 and 8	Every 28 days
cef	5-Fluorouracil 500 mg/m^2^ IV, days 1 and 8 Epirubicin 60 mg/m^2^ IV, days 1 and 8 Cyclophosphamide 75 mg/m^2^ PO, days 1–14	Every 28 days
fac	5-Fluorouracil 500 mg/m^2^ IV, day 1 Doxorubicin 50 mg/m^2^ IV, day 1 Cyclophosphamide 500 mg/m^2^ IV, day 1	Every 21 days

IV = intravenous; PO = orally.

**Table II:** Sample case scenario

In the following case scenarios, while more than one treatment option might be appropriate for patient discussion, please assume the patient has informed you that she does *not* want to be part of any clinical trial and wishes *you* to make the decision about her treatment. Please tick off the best treatment option. Please circle more than one answer if you believe 2 or more of the options given are entirely equivalent in terms of *efficacy* in that particular case. (See [Table I] for definitions of treatment regimens described)
An otherwise healthy 59 y.o. woman presents post-mastectomy with ER +ve, PR +ve, grade II, 1.5 cm infiltrating duct carcinoma with focal tumour necrosis but no LVI or perineural involvement. *HER2*/*neu* overexpression is “positive.” Twelve lymph nodes are negative for malignancy. What systemic treatment would you recommend? Hormonal therapy only AC × 4, then hormonal therapy CMF × 6, then hormonal therapy AC × 4, then Taxol × 4, then hormonal therapy CAF or FAC × 6 months, then hormonal therapy MF × 6 months, then hormonal therapy CEF × 6 months, then hormonal therapy Other chemotherapy (please specify regimen and duration: ______________________________), then hormonal therapy What hormonal therapy would you recommend? Tamoxifen An aromatase inhibitor Other (please specify: ______________________________)

**Table III:** Physician characteristics by questionnaire version

	*Version A*	*Version B*
Responded [*n*/*N* (%)]	30/50 (60)	29/49 (59)
Male:female (*n*)	21:9	20:9
Mean training time (years)	14.2	15.1
Area of practice		
Cancer centre	11	11
Academic	7	5
Community	12	13
Mean new patients per month (*n*)	8.7	10.5
Breast-cancer practice (%)	38%	40%
*HER2* routinely available [*n*/*N* (%)]	16/30 (53)	16/29 (55)
